# Trajectories of Dental Caries From Childhood to Young Adulthood: Unsupervised Machine Learning Approach

**DOI:** 10.21203/rs.3.rs-3125821/v1

**Published:** 2023-07-28

**Authors:** Chukwuebuka Ogwo, Steven Levy, John Warren, Daniel Caplan, Grant Brown

**Affiliations:** Temple University; University of Iowa; University of Iowa; University of Iowa; University of Iowa

**Keywords:** Cohort Studies, Machine learning, Dental Caries, Trajectory

## Abstract

**Objective:**

To determine the dental caries trajectories over the life course (from age 9 to 23) using an unsupervised machine learning approach.

**Methods:**

This is a longitudinal study of caries trajectories over a life course using data from 1,382 individuals from the Iowa Fluoride Study birth cohort. The trajectory analysis of caries in the permanent dentition at ages 9, 13, 17 and 23 was performed using the unsupervised machine learning algorithm known as K-means for Longitudinal Data (KmL), a k-means based clustering algorithm implemented in R specifically designed for analyzing longitudinal data. The trajectory grouping was performed by assessing the distances of the individual trajectories from the centroid and the prediction of the “best” partition was performed based on the Calinsky & Harabatz criterion. The number of cluster partitions assessed was 2 to 6. The number of re-runs with different starting conditions for each number of clusters was 20.

**Results:**

The trajectory analysis identified three trajectory groups with 70.5%, 21.1%, and 8.4% of participants in the low, medium, and high caries trajectory groups, respectively. The mean D_2+_MFS counts of the low caries trajectory groups at ages 9, 13, 17, and 23 were 0.23, 0.37, 1.10, and 1.56, respectively. The mean D_2+_MFS counts of the medium caries trajectory groups at ages 9, 13, 17, and 23 were 0.92, 2.09, 6.24, and 9.55, respectively. The mean D_2+_MFS counts of the high caries trajectory groups at ages 9, 13, 17, and 23 were 1.49, 4.80, 12.91, and 22.52, respectively. There were steeper increases in the D_2+_MFS scores of the three trajectory groups between age 13 and 17, with less steep but also strongly positive slopes from age 17 to 23, suggesting that the period from age 13 to 17 is the highest risk period.

**Conclusion:**

There was an increase in the trajectory slopes after age 13 which might be due to changes in risk factors. The next step in this study will be to identify those factors that predict trajectory group membership by modeling their relationships using supervised machine learning techniques.

## Introduction

As a chronic and cumulative disease, dental caries develops over time and can occur at any stage of life if there are susceptible tooth surfaces.^1^ Throughout life, several factors (biological, behavioral, and environmental) continue to impact oral health, supporting the importance of taking a life-course perspective in the study of dental caries. There are very few long-term prospective studies that have attempted to describe patterns of dental caries experience over a life course, probably because of the difficulty in accumulating such data and the complex statistical approaches required to analyze and interpret the data.^2^

Broadbent et al.^3^ conducted a study to describe and define the longitudinal patterns of caries experience in a birth cohort in New Zealand by investigating their developmental caries trajectories from age 5 to age 32 by performing a group-based trajectory analysis using PROC TRAJ with a zero-inflated Poisson (ZIP) model. Only participants who had at least three dental examinations were included in the study and the optimal model was selected based on the Bayesian Information Criterion (BIC) (Broadbent et al., 2008).^3^ A parsimonious, three-group trajectory analysis model was chosen, with 384 participants (40.2%) assigned to ‘group 1’ (low trajectory group), 427 (44.7%) to ‘group 2’ (medium trajectory group), and 144 (15.1%) to ‘group 3’ (high trajectory group). The higher trajectories showed a non-linear pattern (quadratic “S” shape), while the lower trajectory was linear. At age 32, the mean DMFS was 5.4, 18.6, and 42.3 in group 1, group 2, and group 3, respectively.^3^

Another caries trajectory study by Warren et al.^4^ using the birth cohort from the Iowa Fluoride Study (IFS), including only participants who had completed all three dental examinations at ages 9, 13, and 17, used Ward’s hierarchical clustering algorithm to identify three trajectory groups. The study found that the trajectory group 1 (low trajectory group) had 142 participants (35.9%); trajectory group 2 (medium trajectory group) had 145 participants (36.6%); and trajectory group 3 (high trajectory group) had 109 participants (27.5%).^4^

Group-based trajectory analysis is one of the best approaches to describing growth trajectories over a life course because it can be interpreted easily.^5^ The trajectory groupings help simplify the complex longitudinal data by aggregating them into meaningful clusters according to underlying essential features. Handling small numbers of trajectory groups is a lot easier than analyzing several hundred individual trajectories ^5,6^ and the trajectory groupings make the presentation in graphs and tables simpler and more interpretable by researchers, clinicians, and policymakers. Different approaches can be used to perform the group-based trajectory analysis^7,8^, but this study explores the use of an unsupervised machine learning algorithm (K-Means) in performing group-based analysis.^9^

Also, given the very small number of studies on dental cries trajectory, this study seeks to enhance the understanding of the progression of dental caries experience from childhood to adulthood using group-based trajectory analysis. The aim of this analysis was to determine the trajectories of dental caries from age 9 to 23 among Iowa Fluoride Study (IFS) participants.

## Methods

This is a prospective (cohort) study of Iowa Fluoride Study participants recruited from post-partum wards of eight Iowa hospitals between March 1992 to February 1995 and followed up with oral health questionnaires and examinations.^10^ Dental examinations were carried out every 4 to 6 years and oral health questionnaires were sent approximately every six months.^10^ Approval for the Iowa Fluoride Study was obtained from the University of Iowa Institutional Review Board for all components and procedures of the study. Informed consent was obtained from the participants prior to the examinations and questionnaires during age 23 assessments, with assent obtained at ages 13 and 17. Consent also was obtained from the participants’ parents for all ages to children’s age 17. This trajectory analysis included only participants who had completed the dental exams for at least 2 out of the 4 ages (ages 9, 13, 17, and 23).

Dental Caries Trajectory Groups (Clusters) were defined as subject-specific groups of observations (subject-specific D_2+_MFS counts) clustered based on certain characteristics to identify distinct patterns within the individuals’ observations. That is, subject-specific D_2+_MFS count trajectories were grouped based on certain criteria to form clusters with distinct characteristics. Details were described in the next section.

### Statistical Analysis

The data for this study were extracted from the IFS database and all statistical analysis was performed in R (Version 4.1.3).^12^ Descriptive statistics (frequency, mean, median, maximum, minimum, quartiles, and interquartile range) were calculated for the individual level and trajectory group-based variables. The trajectory analysis was conducted using an unsupervised machine learning algorithm - K-means for Longitudinal Data (KmL)^10,11^ - which is a K-means- based clustering algorithm specifically designed for analyzing longitudinal data.^9,13^ This algorithm can deal with missing values and has an automated mechanism of re-running the algorithm while varying the initiation condition and/or the selection of clusters.

We determined the trajectory group membership using an algorithm that incorporated both the Euclidean and longitudinal data distance (Frechet distance) and handling the missingness with Gower’s distances.^11^ The “best” partitions to explain the data set were determined by running the algorithm through three sets of criteria – Calinski-Harabasz,^11^ Ray-Turi^12^ and, Davies -Bouldin^13^. The best partition was selected based on the Caliński - Harabasz criterion. The higher the Caliński - Harabasz criterion score, the better the selected partition. However, the other criteria were defined to better understand the overall model performance. The number of cluster partitions assessed was 2 to 6 which means that Kml algorithm must search partitions with 2, then 3, up to 6 clusters. The number of re-runs with different starting conditions for each number of clusters was 20. The selected partitions then were visualized and exported with the help of the graphical interface in the KmL package.^11^ Detailed descriptive statistics (frequency, mean, median, mode, maximum, minimum, quartiles, and interquartile range) were calculated for each of the dental caries trajectory groups’ D_2+_MFS counts at the 4 exam periods. We performed sensitivity analyses to compare the results of those individuals who completed all four dental exams versus at least three of the four dental exams versus at least two of the four dental exams.

## Results

The numbers of IFS participants who completed the dental examinations at ages 9, 13, 17, and 23 were 629, 550, 444, and 334, respectively. There was favorable inter-examiner reliability, with kappa statistics of 0.74, 0.70, 0.74, and 0.83 at ages 9, 13, 17, and 23, respectively.

There were 789 participants who completed at least one of the four examinations, but 560 participants (71% of the sample) fulfilled the inclusion criterion for the trajectory analysis by having at least two dental exams. About 51% of the 560 participants were female and 49% were male. About 14.3% of the subjects’ 2007 family income levels were below $40,000, 18.1% were $40,000 to $59,999, 20.1% were $60,000 to $79,999, and 47.5% were $80,000 and above. Up to 11.5% of the subjects’ mothers had a high school diploma or lower, 15.5% had attended some college, 22.6% had a 2-year college degree, 29.9% had a 4-year college degree, and 20.5% had some form of an advanced degree. About 12.9% of participants were in the lower, 34.8% in the middle, and 52.3% in the higher SES group.

### Trajectory analysis

**Figure 1:**
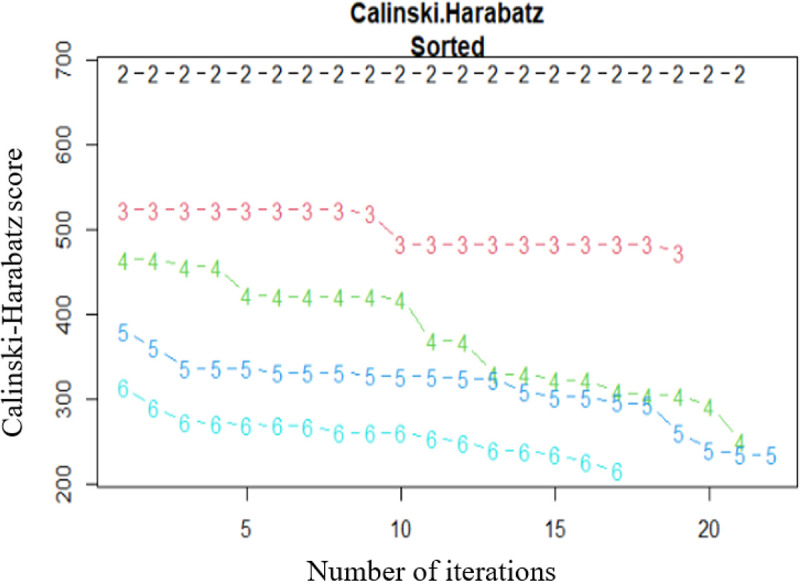
Plot showing the Calinski-Harabatz scores for the different numbers of trajectories. This is a plot of the Calinski-Harabatz score versus the number of iterations for each of the possible trajectory groups. **Note:** Higher the Calinski-Harabatz score indicates better performance of the trajectory group.

Based on the Caliński-Harabasz criterion, the K-means for longitudinal data (KmL) algorithm found two trajectory groups (Caliński-Harabasz score = 684.96) to be the optimal number of partitions, followed by that of the three trajectory groups (Caliński-Harabasz score = 524.13) (Fig. 1). However, we chose the three trajectory groups to be the optimal number of trajectories for this study based on both the Caliński-Harabasz criterion and clinical relevance, in order to capture both medium caries and high caries groups. This will help prevent the loss of potentially clinically relevant information about individuals with moderate levels of caries. The findings of both the two and the three trajectory group classifications are reported.

### 1. Two trajectory group analysis

**Figure 2:**
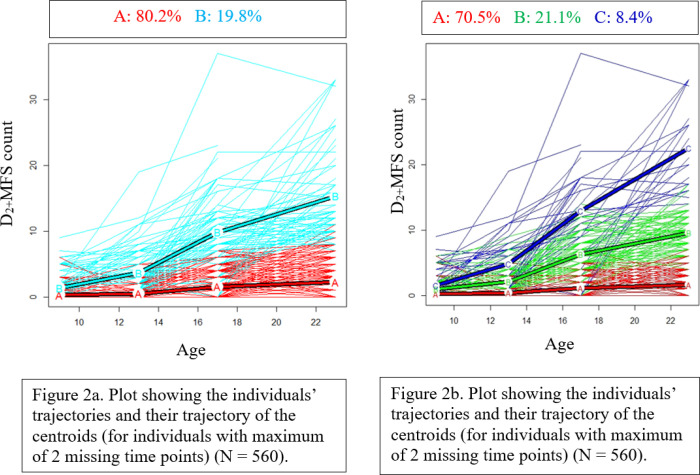
Plot showing the clusters and individual trajectories for two trajectory groups (Left) and three trajectory groups (Right) (for individuals with a maximum of 2 missing time points). **Note:** The thick blue line represents the trajectory of the centroids for participants with high caries trajectory, the thick green line represents the trajectory of the centroids for participants with medium caries trajectory, and the thick red line represents the trajectory of the centroids for participants with low caries trajectory. The thin colored lines represent the trajectories of each individual.

Figure 2a shows a graphical representation of the two trajectory groups and the percentages of individual trajectories in each group. Four hundred and forty-nine (80.2%) of the individual trajectories were in trajectory group A (low caries trajectory group) and 111 (19.8%) in trajectory group B (high caries trajectory group). Table 1 summarizes the mean D_2+_MFS at ages 9, 13, 17, and 23 for each of the trajectory groups. The mean D_2+_MFS counts at ages 9, 13, 17, and 23 for trajectory group A were 0.27, 0.47, 1.56, 2.25, respectively, and the mean D_2+_MFS counts at ages 9, 13, 17, and 23 for trajectory group B were 1.32, 3.64, 9.80, 15.33, respectively.

Also, sensitivity analyses of two trajectory groups comparing results of those individuals who completed all four dental exams versus at least three of the four dental exams versus at least two of the four dental exams showed very similar patterns in terms of the percentages of individuals in trajectory group A (“4-exam group” (80.9%), “3+-exam group” (80.5%) and the “2+-exam group” (80.2%)), however, their total sample sizes differ significantly (“4-exam group” (560), “3+-exam group” (447) and the “2+-exam group” (282)). (Appendix II)

### 2. Three trajectory group analysis

Figure 2b shows a graphical representation of the three trajectory groups and the percentages of individual trajectories in each group. Three hundred and ninety-five (70.5%) of the individual trajectories were in trajectory group A (low caries trajectory group), 118 (21.1%) in trajectory group B (medium caries trajectory group), and 47 (8.4%) in trajectory group C (high caries trajectory group). Table 1 summarizes the mean D_2+_MFS at ages 9, 13, 17, and 23 for each of the trajectory groups. The mean D_2+_MFS counts at ages 9, 13, 17, and 23 for trajectory group A were 0.23, 0.37, 1.10, and 1.56, respectively. The mean D_2+_MFS counts at ages 9, 13, 17, and 23 for trajectory group B were 0.92, 2.09, 6.24, and 9.55, respectively. The mean D_2+_MFS counts at ages 9, 13, 17, and 23 for trajectory group C were 1.49, 4.80, 12.91, and 22.52, respectively.

Sensitivity analyses of three trajectory groups comparing results of those individuals who completed all four dental exams versus at least three of the four dental exams versus at least two of the four dental exams showed generally similar patterns. The percentage of individuals in trajectory group A and B in the “4-exam group” were 80.9% and 19.1%, respectively. The percentage of individuals in trajectory group A and B in the “3+-exam group” were 80.5% and 19.5%, respectively. The percentage of individuals in trajectory group A and B in the “2+-exam group” were 80.2% and 19.8%, respectively (Appendix I). Also, the sensitivity analyses of two trajectory groups comparing results of those individuals who completed all four dental exams versus at least three of the four dental exams versus at least two of the four dental exams showed slightly varying patterns. The percentage of individuals in trajectory group A, B and C in the “4-exam group” were 73.0%, 21.6% and 5.3%, respectively. The percentage of individuals in trajectory group A, B and C in the “3+-exam group” were 70.5%, 21.1% and 8.4%, respectively. The percentage of individuals in trajectory group A, B and C in the “2+-exam group” were 67.6%, 24.6% and 7.8%, respectively (Appendix II). At the individual level, there were fluctuations in the individual trajectories. Therefore, not all the individual trajectories remained monotonically in the same starting “trajectory group” from age 9 to 23. Some individuals’ trajectories crossed each other as they move from age 9 to 23.

## Discussion

As a chronic infectious disease, the public health impact of dental caries is significant and can last through people’s lifetimes if not prevented and/or treated. There are no known studies that have attempted to analyze the trajectory of dental caries from childhood to early adulthood and/or the predictors of the trajectory group membership using an ML approach.

For the trajectory analysis, 560 participants fulfilled the inclusion criteria and were included in the study. Based on a combination of clinical relevance and the Calinsky-Harabatz score, we found three to be the optimal number of trajectory groups for our data. This is the same as for Warren et al.^4^ and Broadbent et al.^3^ who also found three was the optimal number of trajectory groups for ages 9 to 17 within the Iowa Fluoride Study (IFS) and ages 5 to 32 within the New Zealand cohort study, respectively. However, the distribution of the participants across the trajectory groups differed across the three studies, probably due to differences in the samples’ demographics, such as age and socioeconomic status, as well as fluoride, dietary and other factors. This study had 70.5% of participants in the low caries trajectory (group A), which was much higher compared to the 40.2% in Broadbent et al.’s^4^ study and 36.0% in Warren et al.’s^3^ study. Conversely, the percentages of individuals in the medium and high caries trajectory groups were lower in our study (21.1% and 8.4%, respectively) compared to Broadbent et al.’s^4^ study (44.7% and 15.1%, respectively) and Warren et al.’s^3^ study (37.0% and 28.0%, respectively).

There were steeper increases in the D_2+_MFS scores of the three trajectory groups between age 13 and 17, with less steep but also strongly positive slopes from age 17 to 23, suggesting that the period from age 13 to 17 is the highest risk period. Across the different ages, the mean D_2+_MFS counts of the low caries trajectory group were much lower than (about 50% of) the overall sample mean D_2+_MFS counts, while the mean D_2+_MFS counts of the medium trajectory group were about twice as high or more than the overall mean D_2+_MFS counts, and for the high caries trajectory they were about 3 to 4.5 times the means. Also, the low caries trajectory group’s mean D_2+_MFS counts stayed consistently low and the cumulative increase over 14 years (from age 9 to 23) was 1.33 surfaces, or an average of less than 0.1 surfaces per year. In contrast, the middle caries group’s mean increased by 1.27 surfaces at age 13, 4.15 more to age 17, and 3.31 more to age 23, with a cumulative increase of 8.63 surfaces (or more than 0.6 surfaces per year). The high caries trajectory group’s mean increased 3.31 surfaces to age 13, 8.11 more to age 17, and 9.61 more to age 23, with a cumulative increase of 21.03 surfaces (or more than 1.5 surfaces per year). Also, the mean counts for the high caries trajectory were uniformly more than twice the counts of the medium trajectory group, and the absolute magnitude of the differences continued to increase over time. This could help inform policy changes toward more targeted preventive public health and individual interventions for this highest risk age group (from age 13 to 17), hopefully ultimately reducing the magnitudes of the increases in D_2+_MFS counts of the dental caries trajectory groups and associated treatment costs.

At the individual level, there were fluctuations in the individual trajectories. Some individual trajectories remained monotonically in the same starting “trajectory group” from age 9 to 23, while some fluctuated. These fluctuations at the individual level might have been due to factors such as reversal and underdiagnosis at an earlier age due to the lack of use of radiographs for caries assessment. For example, an individual could have had several incipient caries lesions at age 13 and a D_2+_MFS score of 1 and then might have a D_2+_MFS score of 12 at the age 17 assessment because of the progression of the non-cavitated caries to cavitated caries. This also highlights a limitation of caries diagnostic methods used in the IFS, which relied only on visual and tactile methods for caries diagnosis, especially if the intention is to use the finding for clinical decision-making at the individual level. Other limitations include the limited sample size resulting from attrition of participants over the 23-year study period and lack of diversity of study participants (more than 80% of the study participants were white and high SES).

Our study results could help policymakers understand the impact of the different trajectory groups in terms of average annual caries increments with the low caries trajectory group showing minimal need, the medium trajectory group manageable moderate needs, and the high caries trajectory group very large needs. Our study also showed the impact of ML in the modelling of longitudinal caries patterns from childhood to adulthood. A future research direction would be to perform predictive modelling using supervised machine learning approaches to determine the factors that predict the trajectory group membership. In this way, interventions could be made for those in the high trajectory group to reduce dental caries morbidity, thus reducing costs and other impacts on this group. Also, to improve the quality and generalizability of the models, future research could perform clinical validation with data from other groups, including more diverse sociodemographic and higher caries risk groups.

## Conclusion

Our unsupervised ML model showed changes in the trajectory of cavitated caries over the life course from middle childhood to early adulthood, with a steeper increase in D_2+_MFS scores of all three trajectory groups between age 13 and 17. This could help inform policy changes toward more targeted preventive public health and individual interventions for this highest risk age group (from age 13 to 17). The study further emphasizes the great potential of artificial intelligence modelling an individual’s caries trajectory over a life course.

## Figures and Tables

**Table 1.** Mean D_2+_MFS counts at ages 9, 13, 17, and 23 and the percentage distribution for the trajectory groups (N =560).

		Mean D_2+_MFS counts				Percentage distribution
		Age 9	Age 13	Age 17	Age 23		
Two trajectory groups	Trajectory group A	0.27	0.47	1.56	2.25	80.2%
	Trajectory group B	1.32	3.64	9.80	15.33	19.8%
Three trajectory groups	Trajectory group A	0.23	0.37	1.10	1.56	70.5%
	Trajectory group B	0.92	2.09	6.24	9.55	21.1%
	Trajectory group C	1.49	4.80	12.91	22.52	8.4%
	Overall mean D_2+_MFS count	0.47	1.10	3.20	5.05	

## Data Availability

The data that support the findings of this study are available on request from the corresponding author. The data are not publicly available due to privacy or ethical restrictions. We are currently working to share all original Iowa Fluoride Study/Iowa Bone Development Study data later in 2023 through the dbGaP repository under U01- DE028522.
